# 

**Published:** 1842-11-26

**Authors:** 


### CONTENTS OF No. 48.

BIBLIOGRAPHICAL NOTICES: An Eulogium on William P. Dewees, M.D. Delivered before the Medical Students of the University of Pennsylvania, Novembei 5, 1842. By Hugh L. Hodge, M. D. Professor of Obstetrics, &c. &c., - - - - 755

On the Mutual Relations between Anatomy, Pathology, Physiology, and Therapeutics and the Practice of Medicine. Being the Gulstonian Lectures for 1842. By Marshall Hall, M, D., F. R. S. L. and E., Fellow of the Royal College of Physicians, &c. &c., - - - 758

ANALECTA: Dr. Donné on the Means by which we may detect the fact of Masturbation in young children, - - - - - 760

Dr. Bouillaud's election to the Chamber of Deputies, - 760

Hysteria, Catalepsy, Metastasis of Hearing, &c., - - 760

Structure of the Lung, - - - - - - - - 762

Aphthæ of the Neck of theUterus, - - - - - - 763

Case of Ununited Fracture of both bones of the leg, treated by friction and the starch bandage, - - - - - - 763

M. Civiale on structure of the Urethra, - - - - - 763

M. Re s us on the structure of the Teeth, - - - - 764

Concrete Naphtaline in Psoriasis, - - - - - - 765

Hydropathy, - - - - - - - - - - - 766

Hints on the Management of Infants, - - - - - 766

Onyxis, - - - - - - - - - - - 768

Dr. James Johnson's Portraits of the Medical Lecturers of London, - - - - - - - - - - - 768

Death of M. Hourmann, - - - - - - - 768

On the Occurrence of Typhus in Old Persons, - - - 768

